# Constructing catalyst knowledge networks from catalyst big data in oxidative coupling of methane for designing catalysts†

**DOI:** 10.1039/d1sc04390k

**Published:** 2021-09-22

**Authors:** Lauren Takahashi, Thanh Nhat Nguyen, Sunao Nakanowatari, Aya Fujiwara, Toshiaki Taniike, Keisuke Takahashi

**Affiliations:** Department of Chemistry, Hokkaido University North 10, West 8 Sapporo 060-8510 Japan lauren.takahashi@sci.hokudai.ac.jp keisuke.takahashi@sci.hokudai.ac.jp; Graduate School of Advanced Science and Technology, Japan Advanced Institute of Science and Technology 1-1 Asahidai Nomi Ishikawa 923-1292 Japan taniike@jaist.ac.jp

## Abstract

Designing high performance catalysts for the oxidative coupling of methane (OCM) reaction is often hindered by inconsistent catalyst data, which often leads to difficulties in extracting information such as combinatorial effects of elements upon catalyst performance as well as difficulties in reaching yields beyond a particular threshold. In order to investigate C_2_ yields more systematically, high throughput experiments are conducted in an effort to mass-produce catalyst-related data in a way that provides more consistency and structure. Graph theory is applied in order to visualize underlying trends in the transformation of high-throughput data into networks, which are then used to design new catalysts that potentially result in high C_2_ yields during the OCM reaction. Transforming high-throughput data in this manner has resulted in a representation of catalyst data that is more intuitive to use and also has resulted in the successful design of a myriad of catalysts that elicit high C_2_ yields, several of which resulted in yields greater than those originally reported in the high-throughput data. Thus, transforming high-throughput catalytic data into catalyst design-friendly maps provides a new method of catalyst design that is more efficient and has a higher likelihood of resulting in high performance catalysts.

## Abstract 2

Catalyst data created through high-throughput experimentation is transformed into catalyst knowledge networks, leading to a new method of catalyst design where successfully designed catalysts result in high C_2_ yields during the OCM reaction.

## Graphical abstract

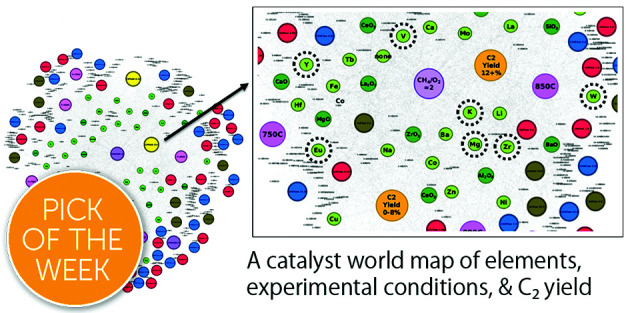

## Introduction

The introduction of catalyst informatics has innovated how catalysts are designed and understood based on the trends and patterns that lie within catalyst data.^1–3^ Catalyst informatics requires consistent and diverse catalyst data, which is becoming more readily available due to developments in catalysis-centered high throughput experiments which are able to produce such series of consistent catalyst big data.^4–6^ While machine learning and data mining have been proven to be effective for extracting knowledge from catalyst data, they are fundamentally limited to expressing the information that is provided by catalyst big data.^7–13^ In particular, it is challenging to design descriptors for representing catalysts during machine learning as catalytic performance is strongly coupled with structural features induced by the interaction of chemical elements in catalysts.^14–16^ In other words, certain chemical elements might have high catalytic performance; however, catalytic performance often increases or decreases depending on how such chemical elements combine with other chemical elements.^17^ Such combinatorial effects are difficult to design as descriptors, thereby still requiring representation of the combination effect of catalysts within catalyst big data. Here, graph theory is proposed as a means to represent the information and knowledge found within catalyst big data where the relationships within catalyst data are represented as complex networks.^18^ Doing so would thus assist in revealing the underlying knowledge in catalyst big data in a comprehensive manner, leading towards a more informed way of designing catalysts.

Catalyst big data for oxidative coupling of methane (OCM) is investigated where OCM aims to directly convert CH_4_ to C_2_H_4_ and C_2_H_6_.^19,20,23^ Big data focused on OCM catalysts are previously collected using high throughput experiments where the dataset consists of 291 catalysts with experimental conditions that result in maximum catalytic performance.^4,5^ If the relationships between chemical element combinations in catalysts and experimental conditions as well as catalytic performance are uncovered, it becomes possible to find key combinations for chemical elements and corresponding experimental conditions that result in high C_2_ yields. Here, the relationships within the OCM catalyst big data are expanded into networks that provide a basis for designing and understanding the OCM reaction from complex networks.

## Methodology

The dataset used in this study is a collection of the OCM data for 291 quaternary catalysts represented by M1–M2–M3/support.^5^ It has two important features that few other catalyst datasets possess. The first feature is the consistency, which arises from the fact that the catalysts were prepared and evaluated by the exactly same methods. When datasets consist of catalyst data collected from multiple references, data inconsistency due to discrepancies in catalyst preparation and evaluation methods is a major obstacle. There are few datasets of this scale that are collected in a consistent manner, and this was achieved through high-throughput experimentation.^4,5^ The second feature is that these 291 catalysts are randomly selected from 36 540 compositions that can be created by combining 28 elements and 9 oxides. The frequency of appearance of individual elements and supports is uniform, without any biases toward known effective compositions, *i.e.* free of sampling bias. The performance of a catalyst as part of a chemical process is sensitive to reaction conditions. Evaluation under specific conditions tends to favor the catalyst that is best suited to those conditions rather than the catalyst that is truly superior. In this dataset, each catalyst is evaluated under 135 reaction conditions with different temperatures and gas compositions, and the data points with the best C_2_ yield are extracted and collected.

## Experimental details

Validation experiments are performed on the catalysts proposed from the analysis of network information. The methods of catalyst preparation and evaluation are exactly the same as those used to create the original dataset.^5^ Briefly, catalysts are prepared based on a wet impregnation method: a specified support (1.0 g) is loaded with precursors of the elements specified as M1–M3 (0.37 mmol for each), followed by drying and calcination at 1000 °C to obtain a catalyst. Support materials and precursors used are the same as those described in the literature.^5^ The OCM performance of the catalysts is acquired using a high-throughput screening instrument developed by some of us.^4,5^ The instrument automatically acquires the performance of 20 catalysts under a pre-programmed set of reaction conditions in a fixed-bed flow reactor configuration. Catalyst beds consist of quartz reaction tubes with an inner diameter of 4.0 mm filled with catalyst powder at a bed height of 10 mm. A gas mixture of a specified composition is simultaneously flowed through 20 catalyst beds heated at a specified temperature, and the composition of the effluent gas is measured using a quadrupole mass spectrometer (QMS) equipped with an auto-sampling system. The catalyst performance is obtained for 135 reaction conditions differing in the temperature and feed volume of CH_4_, O_2_, and Ar, where Ar serves as a carrier gas as well as an internal standard in QMS. As in the original dataset, the data point corresponding to the best C_2_ yield out of the 135 conditions is extracted, which represents the performance of a catalyst.

## Graph theory

Networks of the created datasets are constructed *via* Gephi.^21^ Data from the dataset are extracted and preprocessed to account for graph nodes, edges, and edge weights. Here, graph nodes are objects that represent the catalysts, catalyst supports, corresponding experimental conditions, and the resulting C_2_ yields when tested *via* high throughput experiments. Edges represent the connections shared between two nodes while the edge weight is set to 1. In particular, the following data are extracted for network analysis: atomic elements, catalyst supports, C_2_ yields of the individual catalysts, C_2_ yield groups (0–8%, 8–12%, and 12+%), CH_4_/O_2_ ratio (2, 4, and 6), CH_4_ flow, O_2_ flow, Ar flow, and temperature (700 °C, 750 °C, 800 °C, 850 °C, and 900 °C). Note that in the case of C_2_ yield groups, each catalyst is assigned to a C_2_ yield group according to the individual C_2_ yield produced during the high throughput experiments (*e.g.* catalysts that produce C_2_ yields that are less than 8% belong to the group “C_2_ yield 0–8%”). Based on previous reports, cut-off points are based on catalyst-free OCM which produces a C_2_ yield of 10% with a ±2% range for the “neutral” group (“C_2_ yield 8–12%”).^5^ C_2_ yields that are less than 8% can be seen as yields that are negatively affected by catalytic activity while catalysts with C_2_ yields greater than 12% can be seen as exhibiting higher degrees of catalytic activity. The preprocessed data are then transformed into an undirected graph through the Force Atlas 2 algorithm where node placement is influenced by how often nodes access other nodes (*e.g.* nodes that share many connections are closer to each other within the network).^22^ Note that node sizes and colors are adjusted for visualization purposes.

**Fig. 1:**
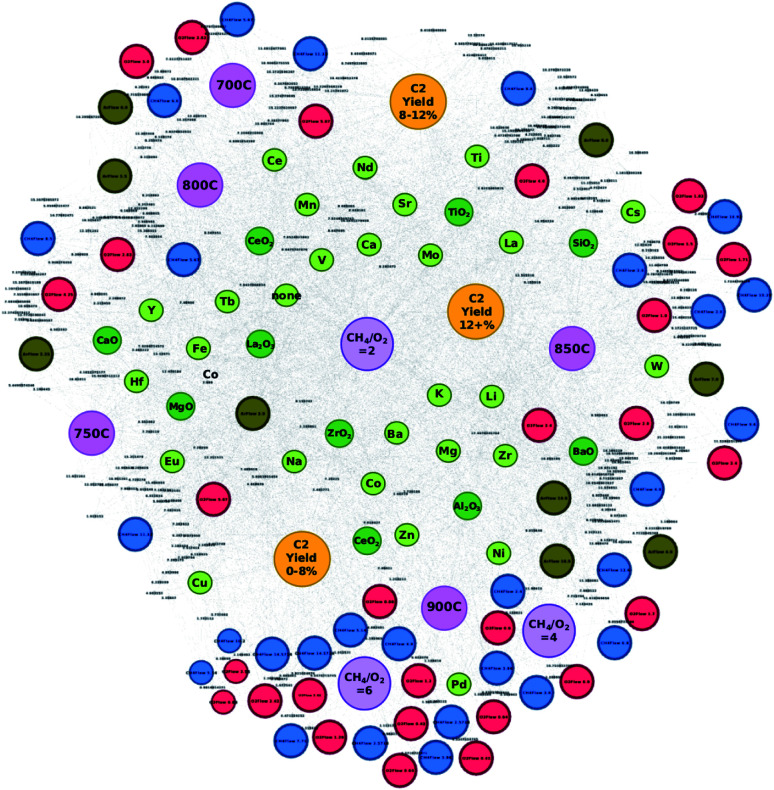
Constructed network consisting of catalyst data with corresponding supports, experimental conditions, and C_2_ yields. Nodes are colored as the following: atomic element (light green), support (dark green), CH_4_ flow (blue), O_2_ flow (red), Ar flow (brown), temperature (pink), CH_4_/O_2_ ratio (purple), and C_2_ yield group (yellow). Individual C_2_ yields are listed by their value. Note that node sizes are adjusted for visualization purposes.

**Fig. 2:**
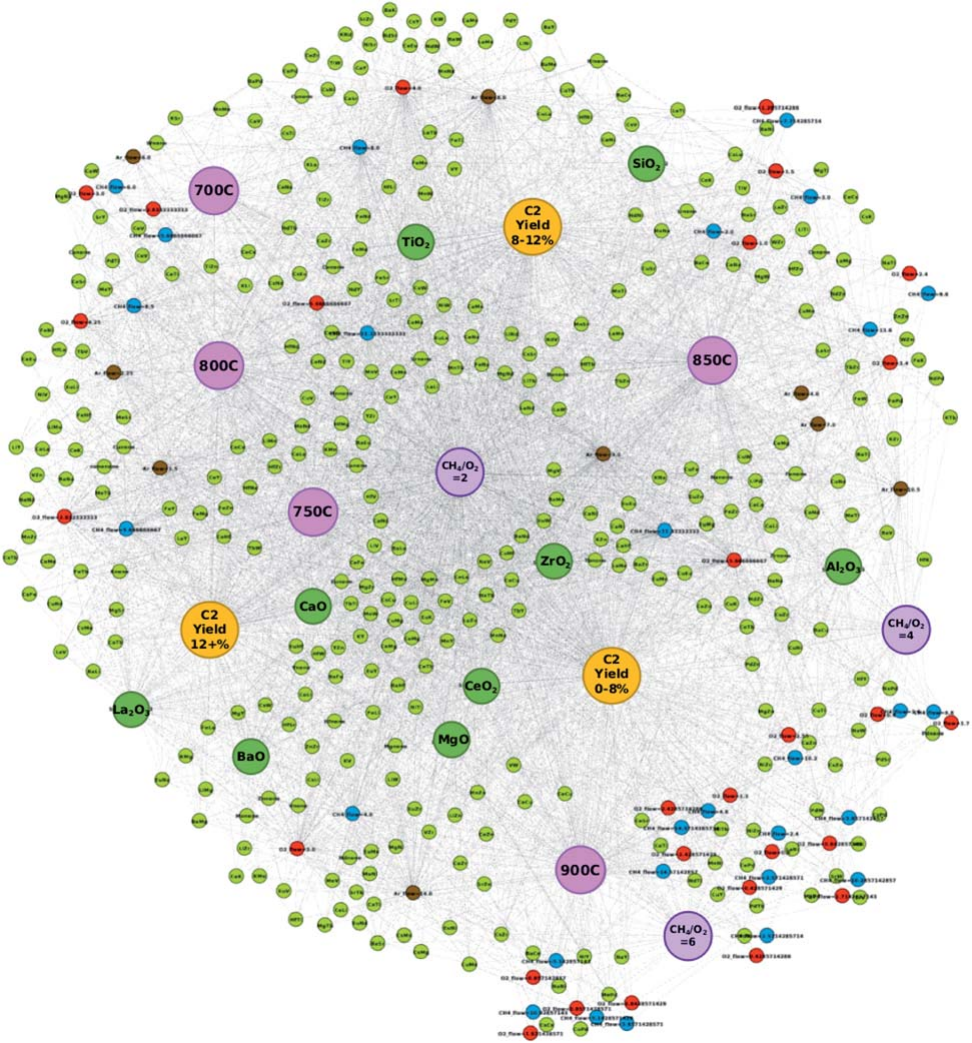
Alternative constructed network consisting of catalyst data with corresponding supports, experimental conditions, and C_2_ yields. Here, elements of the catalysts are represented as element pairs. Nodes are colored as follows: atomic element pair (light green), support (dark green), CH_4_ flow (blue), O_2_ flow (red), Ar flow (brown), temperature (pink), CH_4_/O_2_ ratio (purple), and C_2_ yield group (yellow). Note that node sizes are adjusted for visualization purposes.

Proposed catalysts are designed based on observations and information gathered from the catalyst networks illustrated in Fig. 1 and 2, in particular, elements that either clearly favor the C_2_ yield group “C_2_ yield 12+%” or are found in grey areas between C_2_ yield groups but are found to be closer to the C_2_ yield group “C_2_ yield 12+%”. Additionally, element combinations are chosen based on how often certain element pairs appear near the C_2_ yield group “C_2_ yield 12+%” and how likely they are to pair with particular supports.

## Results and discussion

### Creating an element/experimental condition network

High-throughput experimental data of catalysts used towards the OCM reaction are preprocessed and transformed into a network in order to analyze how various components of data relate to others. By visualizing the data as a network, it becomes possible to make several valuable observations about the catalyst that would otherwise be difficult to obtain when analyzing it in textual format. A network is generated from the collected and preprocessed data using Gephi where individual atomic elements of each catalyst are plotted with their corresponding supports, experimental conditions, and C_2_ yields and can be found in Fig. 1. Catalysts are represented by their atomic elements and supports where each piece is listed individually. For instance, catalyst LiKMn–MgO is represented in the network as nodes “Li”, “K”, “Mn”, and “MgO”; thus, one catalyst is represented by four different nodes. By representing catalysts in this manner, it becomes possible to understand any possible trends present with atomic elements and supports such as tendencies to result in specific levels of catalytic activity or tendencies to pair with a subset of other atomic elements, supports, or particular experimental conditions. Nodes are also colored according to the type of information they represent as follows: atomic element (light green), support (dark green), CH_4_ flow (blue), O_2_ flow (red), Ar flow (brown), temperature (pink), CH_4_/O_2_ ratio (purple), and C_2_ yield group (yellow). Individual C_2_ yields are listed by their value.

From Fig. 1, one can see that nodes representing individual atomic elements found within a catalyst can be found closer to some experimental conditions and C_2_ yield groups rather than others. For example, atomic element nodes such as Pd and Cu are close to the C_2_ yield group “C_2_ yield 0–8%” while atomic element nodes such as Ti and Nd are close to the C_2_ yield group “C_2_ yield 8–12%”. This suggests that these elements have a clearer tendency to result in a particular range of catalytic activity, *e.g.* Pd and Cu tend to result in lower degrees of catalytic activity while Ti and Nd tend to result in a neutral level of catalyst activity when compared to the catalytic activity of other catalysts in this study. In the case of the C_2_ yield group “C_2_ yield 12+%”, it becomes less obvious where the boundaries between the C_2_ yield groups lie. Their location between C_2_ yield groups “C_2_ yield 8–12%” and “C_2_ yield 0–8%” results in many elements being placed in the shared spaces between “C_2_ yield 0–8%” and “C_2_ yield 12+%” and between C_2_ yield groups “C_2_ yield 0–8%” and “C_2_ yield 12+%”. Further analysis of the data reveals that atomic elements that fall within these grey areas between C_2_ yield groups “C_2_ yield 0–8%” and “C_2_ yield 12+%” and between C_2_ yield groups “C_2_ yield 0–8%” and “C_2_ yield 12+%” will result in varying levels of C_2_ yields depending on their companion elements, supports, and experimental conditions. From this, one can understand that elements that fall within these so-called grey areas can be treated as elements whose catalytic performance is influenced by other elements or experimental conditions. Thus, the figure successfully illustrates the importance of combinatorial effects in the design of high-performance catalysts.

Fig. 1 also reveals that certain CH_4_ flow, O_2_ flow, and Ar flow conditions are found to closely associate with particular conditions. For instance, nodes representing the CH_4_ flow, O_2_ flow, and Ar flow tend to congregate around the nodes representing temperature. For example, CH_4_ flows 6.0 and 11.33, O_2_ flows 2.83 and 3.0, and Ar flow 6.0 are found in close proximity to the node representing“700 °C” and, as a set of conditions, are close to node “C_2_ yield 8–12%”, this suggests that these particular experimental conditions are likely to be the conditions that elicit the best catalytic performance of the catalysts that fall within this range. Similarly, the network illustrates that the nodes representing gas flows tend to congregate around temperature nodes where particular temperatures will show closer proximity to certain C_2_ yield groups. Given these observations, one can understand two points: (1) gas flows tend to have share more connections with particular temperatures as seen by their congregation patterns, and (2) temperatures show more connections to some C_2_ yield groups over others. One can therefore treat these gas flow/temperature combinations as sets of conditions that have a stronger correlation with particular C_2_ yields.

While the development of the network illustrated in Fig. 1 helps clarify how different combinations of elements, supports, and experimental conditions relate to others, the combinations that result in C_2_ yields that fall under 8% become strikingly clear. Immediately, one can see that a temperature of 900 °C is strongly related to the C_2_ yield group “C_2_ yield 0–8%” along with CH_4_/O_2_ ratios of 4 and 6. One can also see that a large array of CH_4_ flow and O_2_ flow nodes also exhibit a strong correlation with the C_2_ yield group “C_2_ yield 0–8%” along with atomic elements Cu, Pd, Zn, and Ni. Thus, the network better illustrates elements and supports that associate with conditions that correlate with low C_2_ yields and therefore it may be better to avoid them when designing high-performance catalysts.

Interestingly, transforming catalytic data into a network clarifies the outcomes of choosing different CH_2_/O_2_ ratios. The location of the node representing the CH_4_/O_2_ ratio of 2 within the network reflects how commonly this ratio is involved with the various types of catalysts, supports, and experimental conditions that were tested through high-throughput experimentation. Given its location at the center of the network, one can assume that this particular ratio does not show preference to any particular C_2_ yield outcome, thereby suggesting that other factors may be at play when determining C_2_ yields for the cases where the CH_4_/O_2_ ratio of 2 is involved. Meanwhile, CH_4_/O_2_ ratios of 6 and 4 are clearly close to the C_2_ yield group “C_2_ yield 0–8%”, suggesting that using these particular ratios when designing experiments to test catalysts will likely hinder catalytic performance.

Finally, by analyzing Fig. 1, several so-called “grey zones” are found to appear in areas between neighboring C_2_ yield groups. Various elements and experimental conditions are found in areas where they share equal or similar distances between more than one C_2_ yield group, suggesting that particular elements or experimental conditions may associate with a particular C_2_ yield group depending on the other elements, supports, and experimental conditions that they may be paired with. For instance, elements such as Sr or Cs can lead to C_2_ yields that fall within the C_2_ yield range of 8–12% or lead to a yield greater than 12% depending on what they are coupled with. Similarly, elements such as Zr, Mg, and Ba fall within a grey zone between C_2_ yield ranges of less than 8% and greater than 12%, suggesting that the elements' ability to invoke a higher C_2_ yield may depend on the elements or experimental conditions that they are partnered with. While these grey zones provide insights towards designing catalysts that result in higher C_2_ yields, the pairing effect that occurs between elements is still largely unknown.

From these results, it becomes clear that transforming catalytic data into a network provides a wealth of information regarding how various components affect the C_2_ yield of a given catalyst. Not only can one understand the likely C_2_ yield outcome of using different elements when designing a catalyst, but can also understand which experimental conditions can enhance the catalytic activity of the catalyst in question. Visualizing the data in this manner can therefore improve the efficiency of the catalyst design process and allow researchers to extract knowledge and apply it towards new catalysts and experimental designs.

### Analyzing the effect of element pairing

In order to better understand the effects of element pairing in relation to various experimental conditions and resulting C_2_ yields, the network is redesigned by representing element combinations as element pairs. The catalytic data are preprocessed in the same manner as previously discussed; however, catalysts are represented by the possible element pairs that can be made with the individual elements of the catalyst instead of individual atomic elements. For instance, catalyst LiKMn–MgO, which was previously represented in the network as nodes “Li”, “K”, “Mn”, and “MgO”, is now represented as the following: “LiK”, “LiMn”, “KMn”, and “MgO”. By representing catalysts by their element pairs, the ability to design new catalysts that elicit a high C_2_ yield based on the network visualization becomes possible as it can potentially help clarify positive combinations of elements that may have otherwise fell within the “grey areas” between C_2_ groups as found in Fig. 1. This is in part due to how node placement is determined when constructing the network where node locations are determined by how frequently one piece of data accesses or is accessed by another piece of data within the dataset. By representing the elements of a catalyst as element pairs, it becomes easier to determine which element combinations will likely result in high or low C_2_ yields. Supports, CH_4_ flow, O_2_ flow, Ar flow, CH_4_/O_2_ ratios (2, 4, and 6), temperatures (700 °C, 750 °C, 800 °C, 850 °C, and 900 °C), and C_2_ yield groups “C_2_ yield 0–8%”, “C_2_ yield 8–12%”, and “C_2_ yield 12+%” are also defined as nodes. Nodes are also colored according to the type of information they represent and are colored as the following: atomic element pair (light green), support (dark green), CH_4_ flow (blue), O_2_ flow (red), Ar flow (brown), temperature (pink), CH_4_/O_2_ ratio (purple), and C_2_ yield group (yellow). Edges represent the connections shared between two nodes while the edge weight is set to 1. For the new network, individual C_2_ yield values are excluded in order to focus on the element pair nodes.

Fig. 2 illustrates the new network where elements within a catalyst are represented as their possible pairs. For instance, elements of catalyst LiEuW–ZrO_2_ would be represented as LiEu, LiW, and EuW, respectively, while its support ZrO_2_ is represented separately. By representing the elements in this manner, the pairing effect becomes clearer. For instance, in Fig. 1, element Ba is located within a grey zone between yield groups “C_2_ yield 12+%” and “C_2_ yield 0–8%”. However, when represented as pairs, one can see that element pair BaEu correlates more with the yield group “C_2_ yield 12+%” than with the C_2_ yield group “C_2_ yield 0–8%”. Cases like W also prove to be interesting when comparing the location of nodes between networks. In Fig. 1, the node representing W is found to be closely related to the yield group “C_2_ yield 12+%”. In Fig. 2, W is found to be much more closely related to the yield group “C_2_ yield 12+%” when paired with elements such as Cs, Mo, Hf, and Li. Meanwhile, W more closely relates to the yield group “C_2_ yield 0–8%” when paired with elements Pd and Sr. This therefore illustrates that the catalytic performance of elements is affected by the elements they are paired with, which can improve or worsen the catalytic activity of the catalyst.

Representing elements in this manner also helps dispel preheld ideas that particular elements are considered to be poor. As seen in Fig. 1, the element Pd is strongly associated with the C_2_ yield group “C_2_ yield 0–8%”; however, Fig. 2 illustrates that Pd, when paired with Ti, Ba, or Co, is found to be much more closely associated with the C_2_ yield group “C_2_ yield 8–12%”. The elements Ti, Ba, and Co, in the meantime, are positioned near the C_2_ yield group “C_2_ yield 8–12%” or within the grey zone between C_2_ yield groups “C_2_ yield 12+%” and “C_2_ yield 0–8%”. This suggests that elements that may be considered to traditionally have poor catalytic performance could potentially be improved by pairing with elements that are typically viewed as having good catalytic performance. Furthermore, the network in Fig. 2 helps clarify ambiguity regarding elements that fall within the grey zones between the C_2_ yield groups in Fig. 1. Thus, by looking at these networks, it becomes possible to design new element combinations that may result in C_2_ yields higher than 12% by combining elements and experimental conditions that fall within the vicinity of the C_2_ yield group “C_2_ yield 12+%”.

### Testing designed catalysts based on network visualization

In order to test the efficiency of designing catalysts based on network visualization, 32 catalyst combinations are designed and then tested *via* high-throughput experiments. Atomic element combinations and potential experimental conditions are proposed using the networks illustrated in Fig. 1 and 2. A glance at Fig. 1 shows that atomic elements such as W, Li, K, Mo, and La strongly associate with the C_2_ yield group “C_2_ yield 12+%” while atomic elements such as Ca, V, Mn, and Tb are found in a grey area between C_2_ yield groups “C_2_ yield 8–12%” and “C_2_ yield 12+%”. Given that these elements are involved in designing catalysts that result in various C_2_ yields, a more detailed network like the one shown in Fig. 2 becomes necessary in order to pinpoint element combinations that potentially result in a desired outcome like high C_2_ yield.

An initial glance at Fig. 2 shows that supports BaO, CaO, and La_2_O_3_ are strongly associated with the C_2_ yield group “C_2_ yield 12+%”, suggesting that these supports have a higher likelihood of resulting in C_2_ yields when used experimentally. From there, element combinations that are found close to these supports are analyzed. Closer analysis of Fig. 2 shows that element W, which is found to strongly associate with the C_2_ yield group “C_2_ yield 12+%” in Fig. 1, is also found to be paired with elements that correlate with the C_2_ yield group “C_2_ yield 12+%”. Similar observations are made for elements such as Ca and Tb with pairs such as CaK, CaTi, CaNd, FeTb, MoTb, and TbTi. By listing the atomic elements according to the additional atomic elements they are paired with, it becomes easier to understand which particular combinations of elements may result in a higher C_2_ yield. This can help clarify cases where atomic elements fall within grey zones as the element pairs can clarify which particular combinations of elements will fall under different C_2_ yield groups.

Designing catalysts according to node placements within the networks is further investigated in order to determine the accuracy and efficiency of designing catalysts in this manner. Table 1 lists the first batch of catalysts predicted with this method. Catalysts are designed based on the information visualized in Fig. 1 and 2. Fig. 1 is used to select elements that clearly favor the C_2_ yield group “C_2_ yield 12+%” or are found in grey areas between C_2_ yield groups but also show affinity for “C_2_ yield 12+%”. Fig. 2 is used to not only find combinations of these elements that fall within the vicinity of the C_2_ yield group “C_2_ yield 12+%” as seen in Fig. 1, but also search for any elements that are observed in a sizeable number of element pairs within the “C_2_ yield 12+%” range. Also, element combinations are chosen based on elements that are found to be common in element pairs near a particular support.

**Table 1:** Proposed catalysts based on network information. Variables represent the following: A; element, B; support, *C*; temperature (°C), *D*; CH_4_ flow (mL min^−1^), *E*; O_2_ flow (mL min^−1^), *F*; Ar flow (mL min^−1^), *G*; CH_4_/O_2_ ratio (mol mol^−1^), and *H*; C_2_ yield(%). Note that C_2_ yields correspond to the best yields when individual catalysts are tested under 135 sets of reaction conditions *via* high-throughput experiments

A	B	*C*	*D*	*E*	*F*	*G*	*H*
TiKW	BaO	850	4	2	14	2	16.45
TiCsW	BaO	850	4	2	14	2	17.45
TiTbW	BaO	800	8	4	8	2	17.14
SrHfnone	BaO	850	4	2	14	2	15.01
SrVnone	BaO	850	9.6	2.4	8	4	11.84
SrHfMo	BaO	850	4	2	14	2	13.27
SrMoW	BaO	900	4.8	2	14	2	13.54
SrBaMo	BaO	850	4.8	1.2	14	4	16.81
MoCsLi	BaO	850	4	2	14	2	17.39
MoLiW	BaO	850	4	2	14	2	16.28
MoVW	BaO	900	4.8	1.2	14	4	14.26
MoKW	BaO	850	4	2	14	2	18.36
MoCsZr	BaO	850	4	2	14	2	17.96
CsZrW	BaO	800	4	2	14	2	17.32
KVW	BaO	850	4.8	1.2	14	4	15.01
VWMo	BaO	900	4.8	1.2	14	4	14.25
KYMo	BaO	850	4	2	14	2	17.60
KYV	BaO	850	4	2	14	2	18.21
EuMgZr	BaO	800	8	4	8	2	18.82
EuHfW	ZrO_2_	850	8	4	8	2	8.05
EuKW	ZrO_2_	800	11.3	5.7	3	2	8.30
BaEuW	ZrO_2_	850	4	2	14	2	15.74
EuVW	ZrO_2_	850	11.3	5.7	3	2	8.32
LiEuW	ZrO_2_	800	4	2	4	2	14.16
EuYW	ZrO_2_	850	11.3	5.7	3	2	7.74
EuCsW	ZrO_2_	850	4	2	14	2	9.13
EuMoW	ZrO_2_	850	8	4	8	2	8.86
EuLiW	ZrO_2_	850	3	1.5	10.5	15	13.68
KVW	MgO	800	6	3	6	2	8.47
TiCeW	TiO_2_	850	8	4	8	2	9.11
TbHfW	La_2_O_3_	700	8	4	8	2	12.09
TbTinone	CaO	700	8	4	8	2	16.65

The catalysts suggested in Table 1 are tested experimentally. Out of the suggested elemental combinations, 23 cases result in a C_2_ yield that can be categorized as “C_2_ yield 12+%”, 8 cases result in a C_2_ yield that can be categorized as “C_2_ yield 8–12%”, and 1 case results in a C_2_ yield that can be categorized as “C_2_ yield 0–8%”. From this, one can see that over half of the suggested elemental combinations result in high C_2_ yields; more specifically, 70% of the catalysts produced a C_2_ yield of 12% or greater when tested *via* high throughput experiments. In particular, catalysts EuMgZr–BaO, MoKW–BaO, and KYV–BaO result in C_2_ yields (%) of 18.82, 18.36, and 18.21, respectively, while catalysts MoCsZr–BaO, KYMO–BaO, TiCsW–BaO, MoCsW–BaO, CsZrW–BaO, and TiTbW–BaO resulted in C_2_ yields (%) of 17.96, 17.60, 17.45, 17.39, 17.32, and 17.14, respectively. One can therefore understand that using the constructed network to represent catalysts and experimental conditions with their respective yields can help increase the likelihood of designing a catalyst with higher C_2_ yields.

**Fig. 3:**
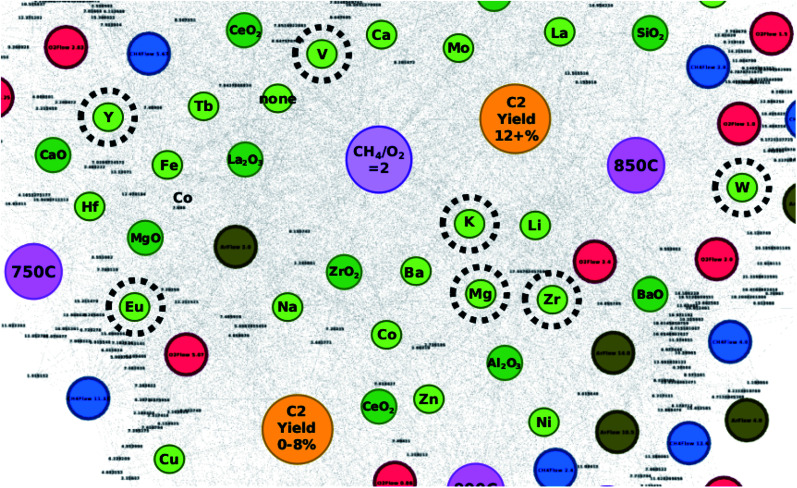
Locations of select elements (circled in black) within the catalyst network. Note that the circled elements are found within the proposed catalysts that resulted in C_2_ yields of 18% when validated *via* experiments.

The elements of these catalysts are compared against their locations within the created networks in order to better understand the reliability of network-based catalyst design. To start with, the elements that make up the catalysts that result in C_2_ yields of 18% – Eu, Mg, Zr, Mo, K, W, Y, and V – are highlighted in Fig. 3 which shows that these elements often fall within a grey area found between C_2_ yield groups “C_2_ yield 12+%” and “C_2_ yield 0–8%”. Elements that make up the catalysts that result in C_2_ yields of 17% – Mo, Cs, Zr, K, Y, Ti, W, Li, and Tb – are also not only found within the grey areas between C_2_ yield groups “C_2_ yield 12+%” and “C_2_ yield 0–8%”, but in some cases are also between C_2_ yield groups “C_2_ yield 12+%” and “C_2_ yield 8–12%”. From this, one can come to the understanding that the efficiency of these elements is affected by the elements that they are paired with.

**Fig. 4:**
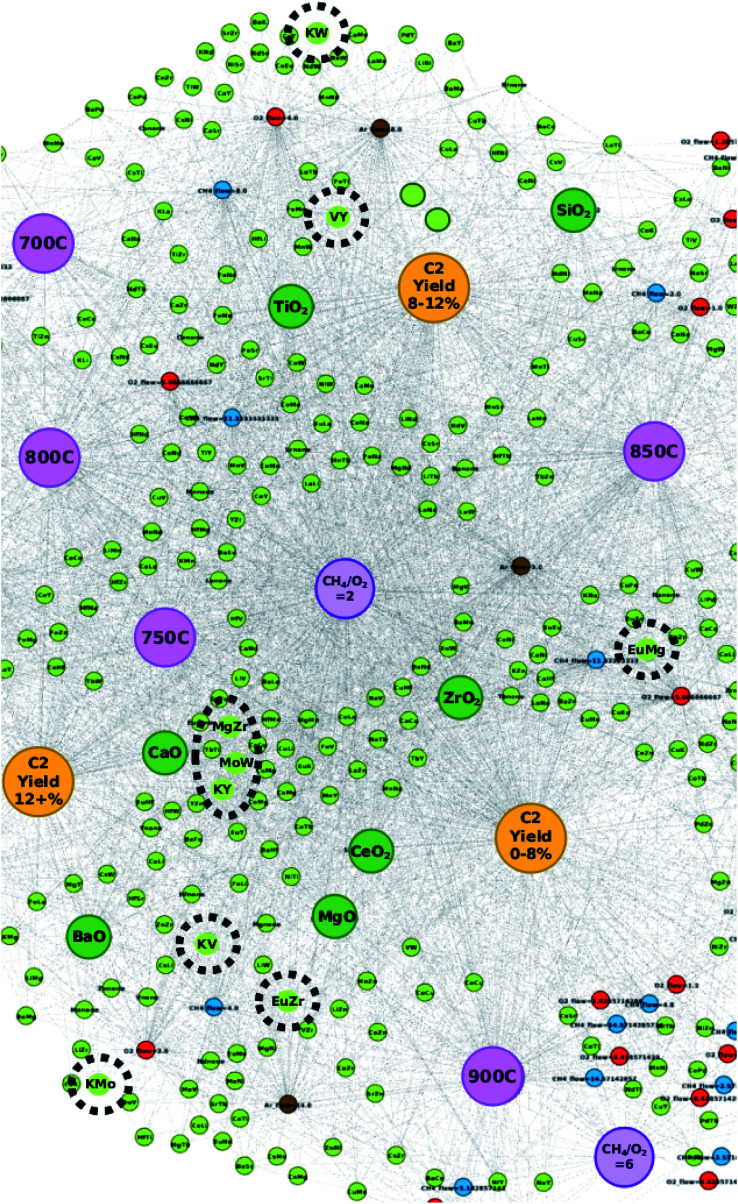
Locations of element pairs (circled in black) for catalysts EuMgZr–BaO, MoKW–BaO, and KYV–BaO, which are found to have a C_2_ yield of 18%.

Fig. 4 illustrates where these elements can be found in relation to the C_2_ yield groups when represented by their element pairs as listed in Table 2. By representing the data in this manner, the particular pairs of elements that result in high C_2_ yields become clearer. For instance, in the case of proposed catalyst “EuMgZr–BaO”, the element pair “EuMg” is found closer to the C_2_ yield group “C_2_ yield 0–8%” while element pairs “MgZr” and “EuZr” are found closer to the C_2_ yield group “C_2_ yield 12+%” and in the grey area between groups “C_2_ yield 12+%” and “C_2_ yield 0–8%”, respectively. Here, one can see that while “EuMg” may be more associated with catalysts that result in C_2_ yields that are low, their combination with element Zr improves the C_2_ yield (as seen by the placements of “MgZr” and “EuZr”). This effect is also seen with proposed catalysts MoKW–BaO and KYV–BaO, where element pairs “MoK” and “VY” share association with the C_2_ yield group “C_2_ yield 8–12%” and the remaining element pairs are found near the C_2_ yield group “C_2_ yield 12+%”. By studying the locations of these element pairs, it becomes possible to not only improve the efficiency of a designed catalyst by choosing element combinations that strongly associate with high C_2_ yields but also can potentially improve the efficiency of catalysts with poor performance by selectively replacing elements with other elements that result in higher catalytic performance.

**Table 2:** Proposed catalysts of Table 1 represented by their element pairs

Proposed catalyst	Element pair 1	Element pair 2	Element pair 3
EuMgZr–BaO	EuMg	EuZr	MgZr
MoKW–BaO	MoK	MoW	KW
KYV–BaO	KY	KV	VY
MoCsZr–BaO	MoCs	MoZr	CsZr
KYMo–BaO	KY	KMo	KMo
TiCsW–BaO	TiCs	TiW	TiW
MoCsLi–BaO	MoCs	MoLi	CsLi
CsZrW–BaO	CsZr	CsW	ZrW
TiTbW–BaO	TiTb	TiW	TbW
SrBaMo–BaO	SrBa	SrMo	BaMo
TbTi–CaO	TbTi	Tb	Ti
TKW–BaO	TK	TW	KW
MoLiW–BaO	MoLi	MoW	LiW
BaEuW–ZrO_2_	BaEu	BaW	EuW
SrHf–BaO	SrHf	Sr	Hf
KVW–BaO	KV	KW	VW
MoVW–BaO	MoV	MoW	VW
LiEuW–ZrO_2_	LiEu	LiW	EuW
EuLiW–ZrO_2_	EuLi	EuW	LiW
SrMoW–BaO	SrMo	SrW	MoW
SrHfMo–BaO	SrHf	SrMo	HfMo
TbHfW–La_2_O_3_	TbHf	TbW	HfW
KVW–MgO	KV	KW	VW
SrV–BaO	SrV	Sr	V
EuCsW–ZrO_2_	EuCs	CsW	CsW
TiCeW–TiO_2_	TiCe	TiW	CeW
EuMoW–ZrO_2_	EuMo	EuW	MoW
EuVW–ZrO_2_	EuV	EuW	VW
EuKW–ZrO_2_	EuK	EuW	KW
EuHfW–ZrO_2_	EuHf	EuW	HfW
EuYW–ZrO_2_	EuY	EuW	YW

A second batch of catalysts are then proposed and are presented in Table 3. Combinations are chosen based on observations made with previous results to explore element combinations that were not initially present in the data. Out of the second set of proposed catalysts, 7 are found to produce C_2_ yields that fall within the category of “C_2_ yield 12+%” while the remaining two produce C_2_ yields that fall within the category “C_2_ yield 8–12%”. No catalysts produce yields that would fall within the C_2_ yield category “C_2_ yield 0–8%”. Thus, one can see that using the created networks to design catalysts in an informed manner can help decrease time and resources spent on catalyst development and testing while also have a higher chance of successfully returning a C_2_ yield that is considered to be high.

**Table 3:** Second batch of proposed catalysts. Variables represent the following: A; element, B; support, *C*; temperature (°C), *D*; CH_4_ flow (mL min^−1^), *E*; O_2_ flow (mL min^−1^), *F*; Ar flow (mL min^−1^), *G*; CH_4_/O_2_ ratio (mol mol^−1^), *H*; C_2_ yield(%). Note that experimental C_2_ yields are in reference to C_2_ yields produced when the catalysts are tested *via* high-throughput experiments

A	B	*C*	*D*	*E*	*F*	*G*	*H*
KVEu	BaO	850	4	2	14	2	20.38
VMoEu	BaO	850	4	2	14	2	16.96
KCaMo	BaO	800	4	2	14	2	18.23
KVZr	BaO	850	4	2	14	2	14.8
MgZrCs	BaO	800	4	2	14	2	15.16
MgYZr	BaO	850	4	2	14	2	18.62
KVY	CaO	750	11.33	5.67	3	2	11.94
KYMo	CaO	750	8	4	8	2	11.49
LiTiW	BaO	850	4	2	14	2	19.03

**Fig. 5:**
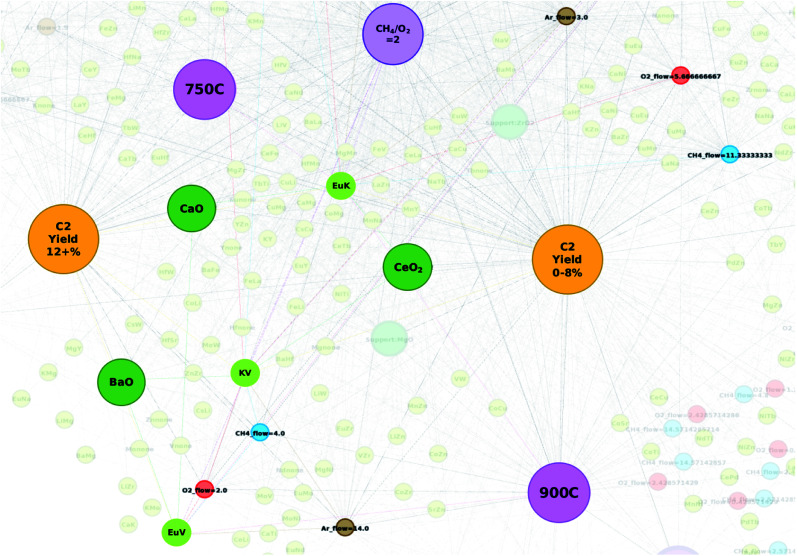
Element pair nodes for proposed catalyst KVEu–BaO and experimental condition nodes that they relate to.

Catalysts KVEu–BaO and LiTiW–BaO are also found to elicit C_2_ yields of 20.38% and 19.03%, respectively, which outperform those of the remaining proposed catalysts and have also not been previously reported. Further analysis is conducted in order to better understand why these combinations may have resulted in such high yields. Fig. 5 illustrates the element pair nodes for proposed catalyst KVEu–BaO that share connections with the nodes for the experimental conditions. Here, one can see that the element pair nodes EuV, KV, and EuK share connections with supports and other experimental conditions that fall around the C_2_ yield groups “C_2_ yield 12+%” and “C_2_ yield 0–8%”. Given that the element pair nodes are located in the grey area between the two C_2_ yield groups, it is likely that the success of these elements is in someway dependent on the supports and gas flows that accompany them. For instance, supports BaO and CaO are seen to have a strong correlation with the C_2_ yield group “C_2_ yield 12+%” while support CeO_2_ strongly correlates with “C_2_ yield 0–8%”. A similar effect is also seen with LiTiW–BaO, where element pairs LiTi and TiW are seen near the C_2_ yield group “C_2_ yield 8–12%” and LiW is found within the grey area between C_2_ yield groups “C_2_ yield 12+%” and “C_2_ yield 0–8%”. Interestingly, the network did not include a case where any of these element pairs are connected with the support BaO. Given that the node for support BaO correlates strongly with the C_2_ yield group “C_2_ yield 12+%”, it is reasonable to believe that pairing the mid-level performing elements with a potentially high-level performing element with a support like BaO can improve the catalytic performance of the proposed catalyst. Further studies, however, are required in order to determine the long-term stability of these catalysts. These results thereby show that targeted design of new catalysts can be carried out more efficiently with the relational information that can be extracted through studying a network representation of catalytic data.

## Conclusion

Transforming catalyst data generated from high-throughput experiments into networks has proven to be beneficial in several ways. To start with, by visualizing the transformation of catalyst data into networks, it becomes easier to understand correlations between atomic elements, their supports, and corresponding experimental conditions in relation to C_2_ yields produced during the OCM reaction. One can see that elements either have clear associations with a particular C_2_ yield group or are found in areas between groups, which suggests that the performance of these so-called “grey-area” elements is influenced by other factors such as the elements they are paired with or other experimental factors such as temperature. The pairing effect of elements on the performance of catalysts is easier to understand when the data are retransformed into a network where catalysts are represented by their possible element pairs. Thirty-two catalysts are then designed using the constructed networks and then tested *via* high-throughput experiments with the aim of producing catalysts that result in high C_2_ yields during the OCM process. Out of the 32 catalysts, 23 are found to result in C_2_ yields greater than 12%, with 9 catalysts resulting in C_2_ yields of 17% or greater. Further analysis of these catalysts shows that elements that are found in grey areas are improved by elements that had correlations with high yield-producing catalysts, thereby demonstrating that catalyst performance can be enhanced through deliberate elemental pairings. Additional catalysts are designed and tested in order to confirm the efficiency of catalyst design *via* a network, where 7 out of the 9 catalysts are found to have C_2_ yields greater than 12%. Two catalysts in particular – KVEU–BaO and LiTIW–BaO – are found to elicit C_2_ yields of 20.38% and 19.03% and have not been previously reported, though long-term stability requires further investigation. Catalyst knowledge networks provide a way to design catalysts based on the relationships provided by catalyst data. In particular, this enables the ability to design highly active OCM catalysts. One can consider that the networks can assist further developments of catalysts, *e.g.* through doping or optimization of composition ratios, by providing information that could potentially lead to the enhancement of catalytic activity. If catalyst big data contains doping and ratio of composition information of catalysts, a further detailed catalyst knowledge network can, in principle, be developed. Thus, by creating networks of catalysts and experimental conditions of data produced *via* high-throughput experiments, catalysts with high performance can be designed in a much more efficient manner with a higher likelihood of success than traditional methods used during the catalyst design process.

## Data availability

Data used to construct the networks presented in Fig. 1 and 2 have been uploaded as part of the ESI.†

## Author contributions

LT and KT conceived the idea for this analysis, determined methodologies, and wrote and reviewed the published work. LT curated catalyst data and applied network-related methods for formal analysis and visualization. TNN, SN, and AF tested designed catalysts in experiment. TT reviewed the published work and provided resources for experimental investigations. KT acquired funding for this published work.

## Conflicts of interest

There are no conflicts of interest to declare.

## Supplementary Material

SC-012-D1SC04390K-s001

SC-012-D1SC04390K-s002

SC-012-D1SC04390K-s003

SC-012-D1SC04390K-s004

SC-012-D1SC04390K-s005

SC-012-D1SC04390K-s006
