# Supplementary figures and images for: Constructing catalyst knowledge networks from catalyst big data in oxidative coupling of methane for designing catalysts
Source: Chem Sci. 2021 Sep 22;12(38):12546–55. doi: 10.1039/d1sc04390k (PMC8494033; doi:10.1039/d1sc04390k)

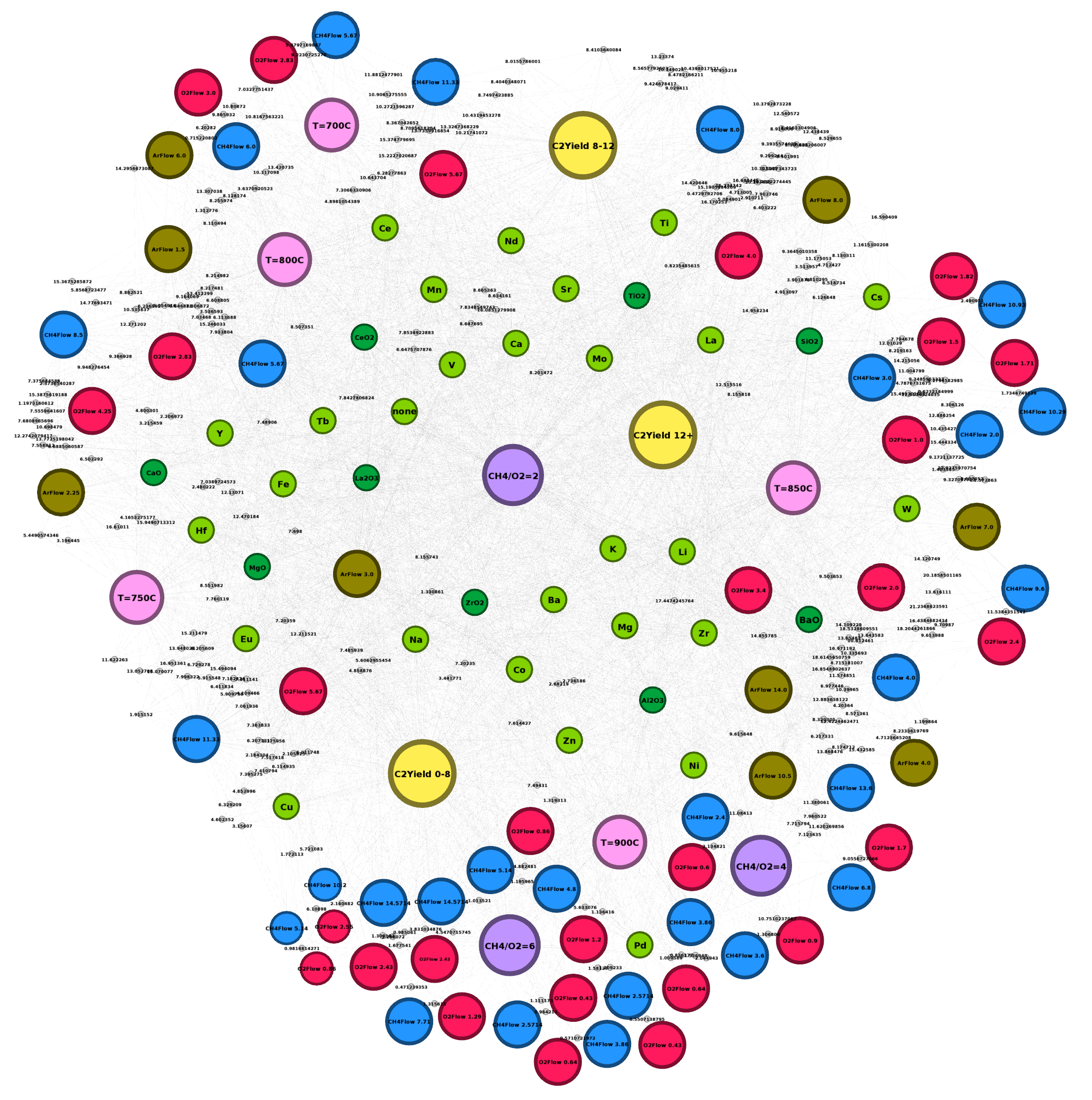

Supplement: SC-012-D1SC04390K-s001 [file SC-012-D1SC04390K-s001.png]

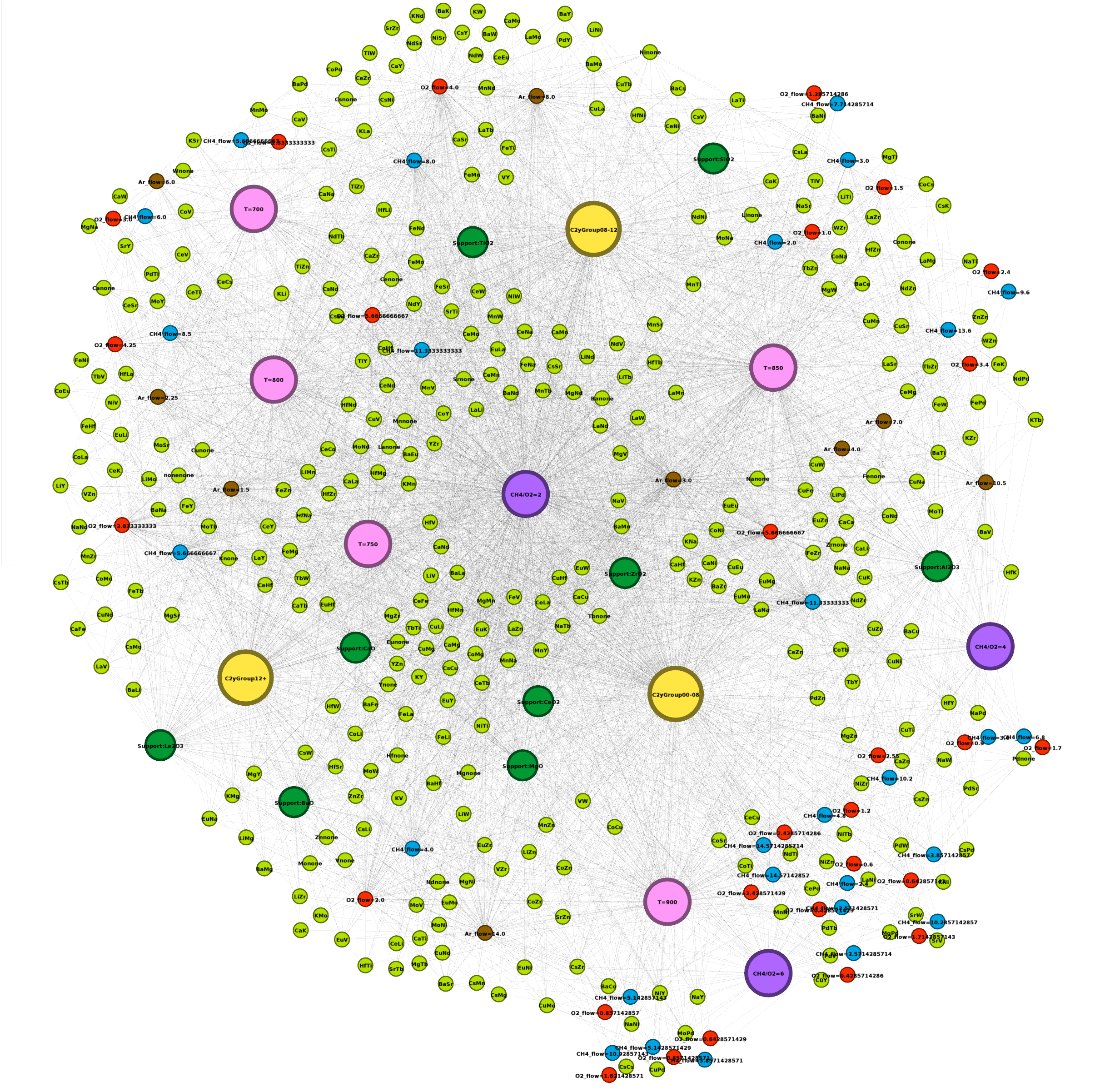

Supplement: SC-012-D1SC04390K-s004 [file SC-012-D1SC04390K-s004.png]
